# Optimal Estimation of Ion-Channel Kinetics from Macroscopic Currents

**DOI:** 10.1371/journal.pone.0035208

**Published:** 2012-04-20

**Authors:** Wei Wang, Feng Xiao, Xuhui Zeng, Jing Yao, Ming Yuchi, Jiuping Ding

**Affiliations:** 1 Key Laboratory of Molecular Biophysics of the Ministry of Education, School of Life Science and Technology, Huazhong University of Science and Technology, Wuhan, Hubei, China; 2 Key Laboratory of Image Processing and Intelligent Control of the Ministry of Education, School of Life Science and Technology, Huazhong University of Science and Technology, Wuhan, Hubei, China; 3 Institute of Life Science, Nanchang University, Nanchang, Jiangxi, China; University of East Piedmont, Italy

## Abstract

Markov modeling provides an effective approach for modeling ion channel kinetics. There are several search algorithms for global fitting of macroscopic or single-channel currents across different experimental conditions. Here we present a particle swarm optimization(PSO)-based approach which, when used in combination with golden section search (GSS), can fit macroscopic voltage responses with a high degree of accuracy (errors within 1%) and reasonable amount of calculation time (less than 10 hours for 20 free parameters) on a desktop computer. We also describe a method for initial value estimation of the model parameters, which appears to favor identification of global optimum and can further reduce the computational cost. The PSO-GSS algorithm is applicable for kinetic models of arbitrary topology and size and compatible with common stimulation protocols, which provides a convenient approach for establishing kinetic models at the macroscopic level.

## Introduction

Ion channels are the pivotal elements of cells, controlling the flow of ions through cell membranes. Voltage-gated channels, for example, are responsible for producing electric signals in excitable cells and thus lay the foundation for life. Different voltage-gated channels exhibit different gating kinetics in response to changes in membrane potentials. For understanding how the channels achieve their functions, it is often necessary to do quantitative analysis of their gating kinetics, because it can provide insights into the functional mechanisms by which they respond to changes of stimulus. Kinetic modeling of ion channels has a long history, dated back to the fifties when Hodgkin and Huxley provided the earliest kinetic models for voltage-gated Na^+^ and K^+^ channels in giant squid axons [1]. Since then, the H-H models have been extensively used in data analysis of cellular electrophysiology. However, with the availability of high resolution data, many ion channels exhibit features beyond the traditional H-H models, such as the multi-stimuli-dependent gating of big-conductance K_Ca_ (BK) channels and the bi-exponential recovery of voltage-dependent Na_V_ channels. As a consequence, more complicated Markov models have been proposed for analysis of ion channel kinetics. Such models usually produce more precise descriptions to the data and provide further insights into the structural and functional mechanisms of the channels. Moreover, the availability of a model will allow one to replicate many properties of the channels such as their responses to various voltage commands, which can be ultimately use to help understand the generations of action potentials in excitable cells [2]. To develop such a model, one faces the inverse problem of Markov modeling, i.e. how to fit a model to data. Depending on the complexity of the models, the problem can be challenging.

It is recently reported by Gurkiewicz and Korngreen [3] that a genetic algorithm(GA) in combination with the Principle Axis technique(PrAxis) was used to globally fit a complicated model to macroscopic responses of voltage-gated channels with a cluster of ten computers in more than a week. The approach appears to work reliably when tested with data simulated from several Hodgkin-Huxley–like and other Markov models of voltage-gated K+ and Na+ channels. Nonetheless, the approach requires extensive computing. Maximum likelihood estimation provides an efficient approach for single-channel analysis [4], [5] and has also been applied more recently to macroscopic data [6]. The method allows for arbitrary stimulation protocols, such as trains of ligand or voltage steps as well as global fitting across multiple experimental conditions. But the method also suffers from intensive computations.

In this study, we aim to develop a whole-cell fitting method, i.e., particle swarm optimization(PSO)-golden section search(GSS) algorithm that is computationally more efficient so that it can be applied for analysis of complicated models with a desktop computer in less than 10 hours. Our tests indicate that the PSO-GSS algorithm can be one or two orders of magnitude faster than the genetic algorithm described by Gurkiewicz and Korngreen [3] (Table S1). To further speed up the algorithm, we also introduce a method for initial estimation of rate constants of the models.

## Results

Before starting a fit, one can set a searching range for PSO-GSS algorithm. In this study, there were two ways for searching the parameter space: the first one to narrow down the searching ranges of parameters derived from the estimated initial values with the boundary factors of 3–5, and the second simply to use the default range of parameters. The first one was expected to accelerate the fitting progress.

**Figure 1:**
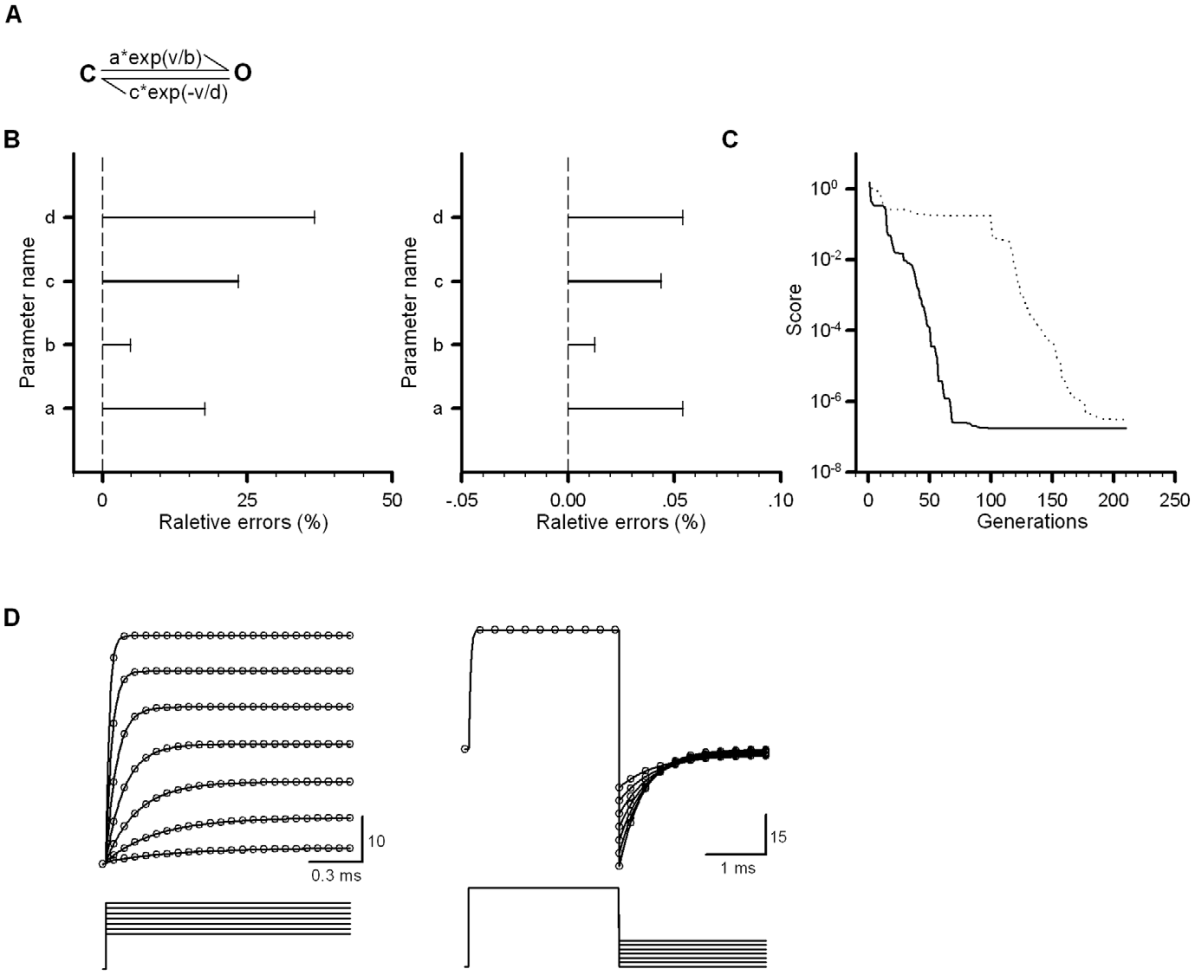
Fit a five-parameter voltage-dependent C-O model to the target current traces. (A) A five-parameter Markov model consisting of a closed state and an open state labeled with the letter C and O, respectively. The forward and backward rate constants separately are a*exp(v/b) and c*exp(−v/d). Here v represents voltage in mV, a and c the pre-exponential factors in ms^−1^ and b and d the exponential factors in mV. The fifth parameter is the channel number N_C_. (B) The errors relative to their target values were obtained by estimation of initial values (left) or by fit (right). (C) Convergence of PSO-GSS with (solid line) or without (dotted line) direct estimation. (D) In this model, target parameters a = 1 ms^−1^, b = 50 mV, c = 1 ms^−1^ and d = 200 mV; the reversal potential of channels V_r_ = 0 mV; the single-channel conductance G = 250 pS and the channel count N_C_ = 1. The empty circles represent the target currents at the various voltages shown under each of current traces, and the solid lines represent fitted currents.

Now we tested a five-parameter C-O model (Figure 1A). With the estimated parameters as we described previously, the maximal relative error was less than 100% of the target values before fitting (Figure 1B, left), and after fitting, it approached zero (Figure 1B, right). During fitting, the generation-course (in a way, time-consuming-course) of scores plotted as a function of generations showed that the optimization took about 100 generations (50 samples per generation) to achieve a score of ∼10^−6^ or less. With default initial parameters (0.5 ms^−1^ for all rates), it took about 200 generations (Figure 1C). At a score ∼10^−6^, the deviation of parameters was <1% from the target values. In Figure 1D, all of the activation (left) and deactivation (right) lines (fits) completely cross the empty circles representing the traces.

A little more complicated example was a nine-parameter model C-O-I (Figure S1A), in which C-O and O-I were separately used to estimate the free parameters. Here we also wanted to point out that some steady-state expressions were often useful in parameter estimation of the incomplete inactivation channels. In the O-I course, we had I_Off_/I_max_ = e*exp(v/f)*τ_i_ and τ_i_(v) = 1/(e*exp(v/f)+g*exp(−v/h)), where I_max_ was the maximal current, I_Off_ the remaining current and τ_i_(v) the inactivation time constants. The values of e*exp(v/f) could be derived from measuring I_Off_/I_max_ and τ_i_(v). In , the maximal relative error of parameters was over 200% before fitting (left), and decreased to about 10^−14^ after fitting (right). With estimated initial values, the optimization took about 1000 generations (100 samples per generation), while with the default values, it took about 1700 generations (Figure S1C). In both cases, the model adequately fitted the activation, deactivation and recovery currents (Figure S1D).

The incorporation of constraints could tremendously reduce number of free parameters. For example, the Kv-like model was composed of four sequential closed states and one open state C_1_-C_2_-C_3_-C_4_-O (or C_4_-O) (Figure S2A). In this model, the forward rate *k* was constrained in a ratio of 4∶3∶2∶1 and the backward rate *k′* in turn in a ratio of 1∶2∶3∶4 if the transition between two consecutive steps was independent. Therefore, total parameters were reduced to five. Because the C_4_-O was identical to four independent C-O courses, it thus had an analytic expression of 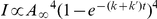 [7], where A_∞_ = *k*/(*k*+*k′*). Fitting the above equation to currents, we could obtain the values of *k+k′* and A_∞_, and thus the *k* and *k′*. Figure S2B showed that the maximal relative error in parameters was about 200% from the target value before fitting (left), and decreased to about 10^−14^ after fitting (right). With estimated initial values, the optimization took about 150 generations (100 samples per generation), while with the default values, it took about 200 generations (Figure S2 C). Again, the resulting model fitted both the activation and deactivation time courses of channel currents perfectly (Figure S2 D and Animation of Kv model). The C_4_-O could be expanded to the C_1_-C_2_-C_3_-C_4_-C_5_-O (or C_4_-C-O) by replacing the last open state O with C_5_-O. Let δ and γ be the forward and backward rates between the C_5_ and O states, respectively, of which both were voltage-independent parameters. In this case, it was difficult to get all of six parameters directly. However, we could get them by the courses C_4_-O or C_5_-O, separately. When transition from C_1_ to C_5_ was much faster than C_5_ to O, e.g. at the higher voltages, we only calculated the slower C_5_-O course to get δ and γ as if C_1_-C_5_ merged into C_5_; when transition from C_1_ to C_5_ was much slower than C_5_ to O, e.g. at the lower voltages, we only calculated the slower C_4_-O course to get the *k* and *k′* as if O merged into C_5_. Remaining parameters should be faster than the calculated ones. With the above estimated initial values, a boundary factor of 3–5 was usually good enough to ensure a parameter range fully covering its target value.

**Figure 2:**
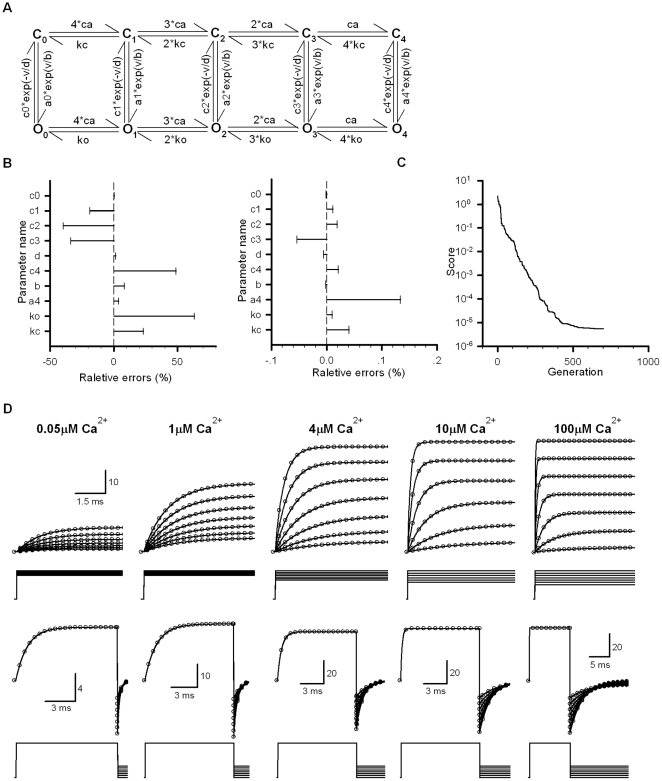
Fit a 11-parameter BK-like C_5_-O_5_ model to the target current traces. (A) A 11-parameter Markov model consisting of five closed states and five open states labeled with the letter C and O, respectively. Each of parameters to be fitted is similar to that we described in Figure 1(A). (B–C) See the description in Figure 1B–C. (D) In this model, the target parameters c0 = 1.8225 ms^−1^, c1 = 1.215 ms^−1^, c2 = 0.855 ms^−1^, c3 = 0.49 ms^−1^, c4 = 0.11 ms^−1^, d = 200 mV, b = 36 mV, a4 = 0.396 ms^−1^, ko = 1.5 µM and kc = 13.5 µM; calcium concentration ca = 0.05, 1, 4, 10 and 100 µM, respectively; the reversal potential of channels V_r_ = 0 mV; the single-channel conductance G = 250 pS and the channel count N_C_ = 1. Four dependent parameters are a0 = 0.001 ms^−1^, a1 = 0.006 ms^−1^, a2 = 0.038 ms^−1^ and a3 = 0.196 ms^−1^. The empty circles denote target currents and the solid lines represent fitted currents. The voltage protocols are placed below each of current traces.

**Figure 3:**
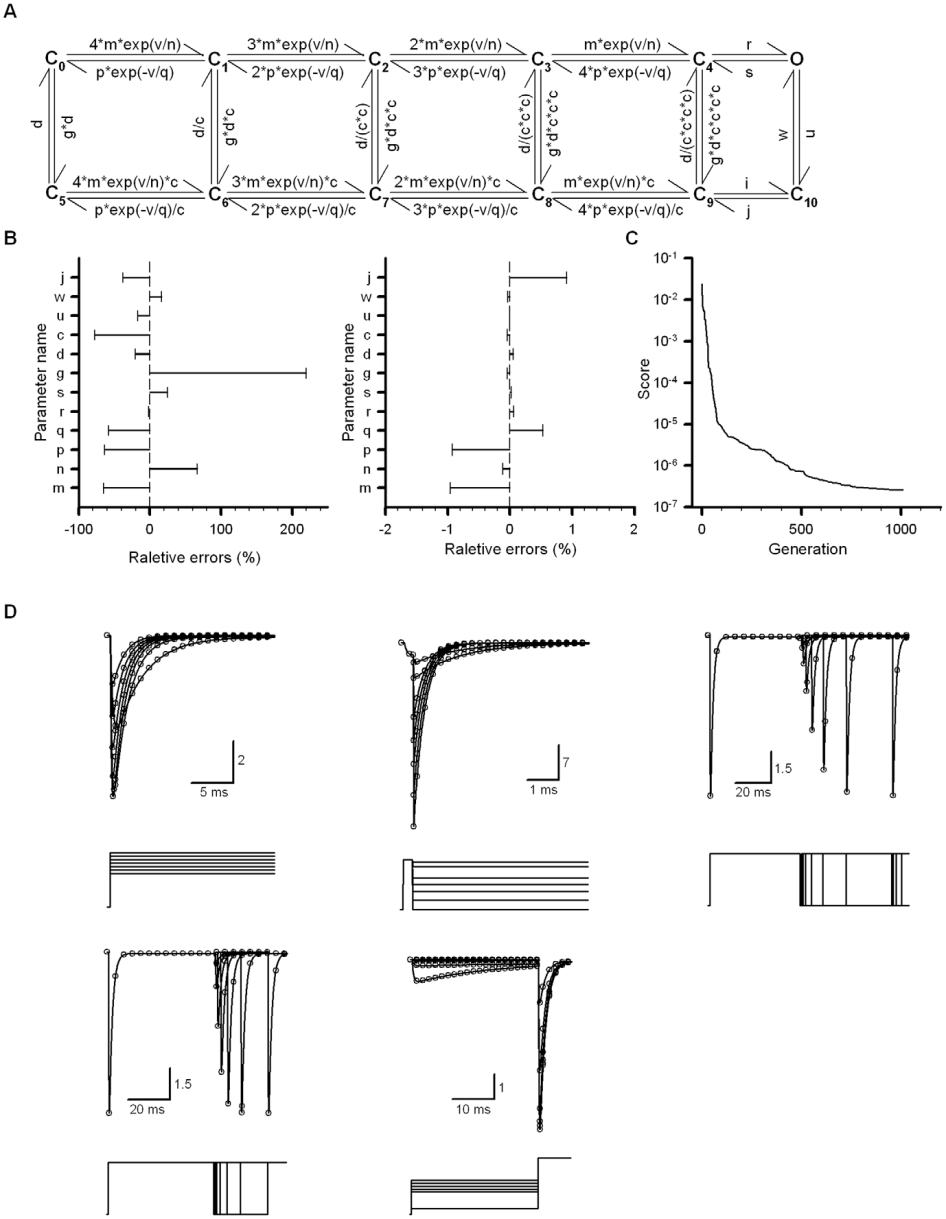
Fit a 13-parameter Na_v_-like C_5_-O-I-CI_5_ model to the target current traces. (A) A 13-parameter Markov model consisting of five closed states (C0, C1, C2, C3 and C4), an open state (O), an inactivation state (C_10_) and five closed-inactivation states (C_5_, C_6_, C_7_, C_8_ and C_9_). Each of parameters to be fitted is similar to that we described in Figure 1(A). (B–C) See the description in Figure 1B–C. (D) In this model, the target parameters m = 288.655598 ms^−1^, n = 12 mV, p = 22.144593 ms^−1^, q = 48 mV, r = 7.5 ms^−1^, s = 2 ms^−1^, g = 0.001, d = 0.5 ms^−1^, c = 4.436203, u = 0.9 ms^−1^, w = 0.006 ms^−1^ and j = 4 ms^−1^; the reversal potential of channels V_r_ = +55 mV; the single-channel conductance G = 250 pS and the channel count N_C_ = 1. A dependent parameter i = 15 ms^−1^. The empty circles denote target currents and the solid lines represent fitted currents. The voltage protocols are placed below each of current traces.

Large conductance calcium- and voltage-dependent potassium (BK) channel had a 10-state allosteric model C_5_-O_5_ named the Monod-Wyman-Changeux (MWC) model [8]. A BK-like C_5_-O_5_ model contained eleven free parameters as shown in Figure 2A. In addition to the constraints we mentioned previously, all the rate constants in this model must obey the microscopic reversibility of cycles, i.e., the product of the rates going clockwise must equal to the product of the rates going anticlockwise. In other words, these constraints of rate constants could be written as follows: Πk_ij_ = Πk_ji_. Here k_ij_ denoted a rate constant of transition between states i→j. To solve this problem, there were three general methods for imposing microscopic reversibility [9]. In this study, we were going to deal with those constraints with a manual way. For a four-state cyclic reaction model, the equation k_12_k_23_k_34_k_41_ = k_14_k_43_k_32_k_21_ allowed one of rate constants to be calculated from the other seven. Therefore, one cycle could reduce one free rate constant. To automatically solve the microscopic reversibility, a factor c (Refer to Figure 3A) was usually introduced into the rate constants of models as we did in the BK-like model [7]. Evidently, there were four cycles in the C_5_-O_5_ to reduce four free rate constants. For estimating initial values of those free parameters, we could discompose the model C_5_-O_5_ according to different Ca^2+^ concentrations. For the intrinsic gating (C_0_-O_0_), we estimated their rate constants at the lowest calcium concentration (e.g. 0.005 µM). For the C_4_-O_4_ rates, we estimated them at the highest calcium concentration (e.g. 100 µM). For other transitions (e.g. C_1_-O_1_, C_2_-O_2_ and C_3_-O_3_), we simply set their initial values in-between. Taking advantage of the detailed balance constraints, we could further reduce the number of free rate constants by four. For the MWC model, its equilibrium probability could be calculated analytically by [8]:
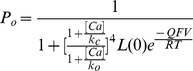
(1)Making use of the relationship, we determined K_O_ and K_C_ by a fit of conductance-voltage (G-V) curves.

Here the maximal relative error in the estimated parameters was about 70% of its target value before fitting (Figure 2B, left), and declined to <0.2% after fitting (Figure 2B, right). In Figure 2C, the estimated way cost about 700 generations (50 samples per generation) to get a good fit. For a complex model with huge amounts of data, the default way was usually hard to give a good fit within hours. To avoid a big computational cost, we could manually or directly fit the element courses decomposed from a complex model to roughly find out its initial values prior to a global fit. Again, both of the activation and deactivation currents at five different calcium concentrations were fitted perfectly (Figure 2D).

A 13-parameter Na_v_-like model denoted as C_5_-O-I-CI_5_ contains five cycles shown in Figure 3A, where CI represented the close-inactivated state [2]. The above model was composing of four kinetic courses: C_0_-C_1_-C_2_-C_3_-C_4_-O (or C_5_-O), O-C_10_ (or O-I), C_9_-C_8_-C_7_-C_6_-C_5_ (or CI_5_) and C_5_-C_0_. The initial parameters of the C_5_-O and O-I courses could be separately estimated by the way described previously. Because C_10_→O was voltage-independent but the channel recovery from inactivation was voltage-dependent, the C_5_-C_0_ course was the only recovering gateway of inactivated channels. We thus determined the rates of C_5_-C_0_ based on the recovery time courses. Here we set the rates of C_9_-C_10_ as those of C_4_-O. The left four cycles automatically satisfied the microscopic reversibility by multiplying a factor c. Additionally, we imposed a constraint i = ruj/(swgc^8^) to satisfy the microscopic reversibility of the rightmost cycle. Here, each of the letters in the above equation, i.e. r, u, j, s, w, g and c, represents the different rate constants in Figure 3A, respectively. In this model, the maximal relative error in parameters was >200% of its target value before fitting (Figure 3B, left), and declined to <1% after fitting (Figure 3B, right). In Figure 3C, the estimated way cost about 1000 generations (50 samples per generation) in less than 10 hours. Similarly, all the activation, deactivation, recovery and steady-state inactivation currents were perfectly fitted (Figure 3D).

**Figure 4:**
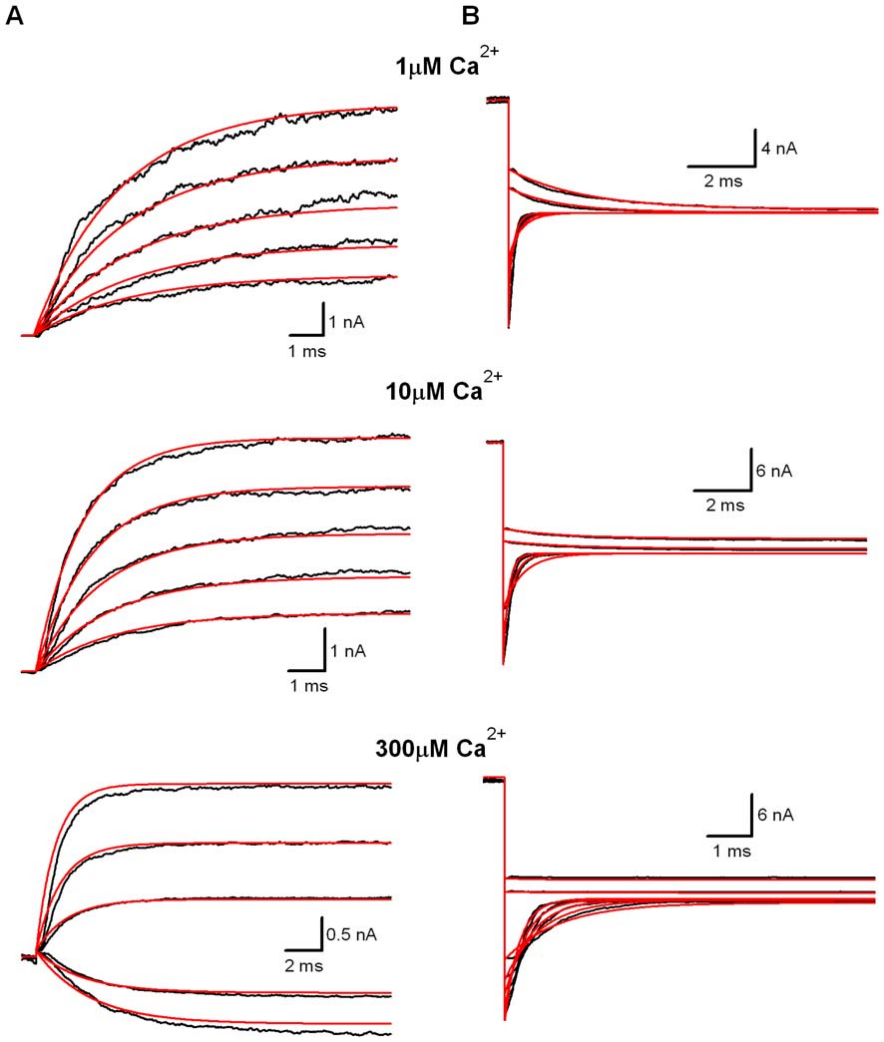
Fit a MWC model C_5_-O_5_ to the macroscopic currents of BK channels from *Xenopus* oocyte. (A) Activation traces of BK currents were recorded from an inside-out patch from a *Xenopus* oocyte injected with cRNA encoding mSlo1 α subunits. Channels were activated by voltage steps ranging from −200 to +200 mV with 10 mV increments from a holding potential of −180 mV with a cytosolic [Ca^2+^]_i_ as indicated. The voltage protocol is not shown here. The red lines were coming from the globally fitting the model C_5_-O_5_ to BK currents by PSO-GSS algorithm. The channel count N_C_ is 314 for 1 µM, 365 for 10 µM and 433 for 300 µM. The different Nc in the same patch is probably coming from the smaller single-channel conductance at the higher Ca^2+^, which will not change the channel kinetics. (B) Deactivation currents were obtained from the same patch as we described in (A). Currents were elicited by voltage steps ranging from −200 to +180 mV with 10 mV increments from a 20 ms-prepulse of +180 mV with a cytosolic [Ca^2+^]_i_ as indicated. The red lines are fits by a PSO-GSS algorithm. The channel count N_C_ is 301 for 1 µM, 354 for 10 µM and 387 for 300 µM. The score σ^2^ is 41.60. All the capacitive currents of 0.15 ms were pre-substituted with straight lines before run and not counted during run. The dash line is zero current.

**Figure 5:**
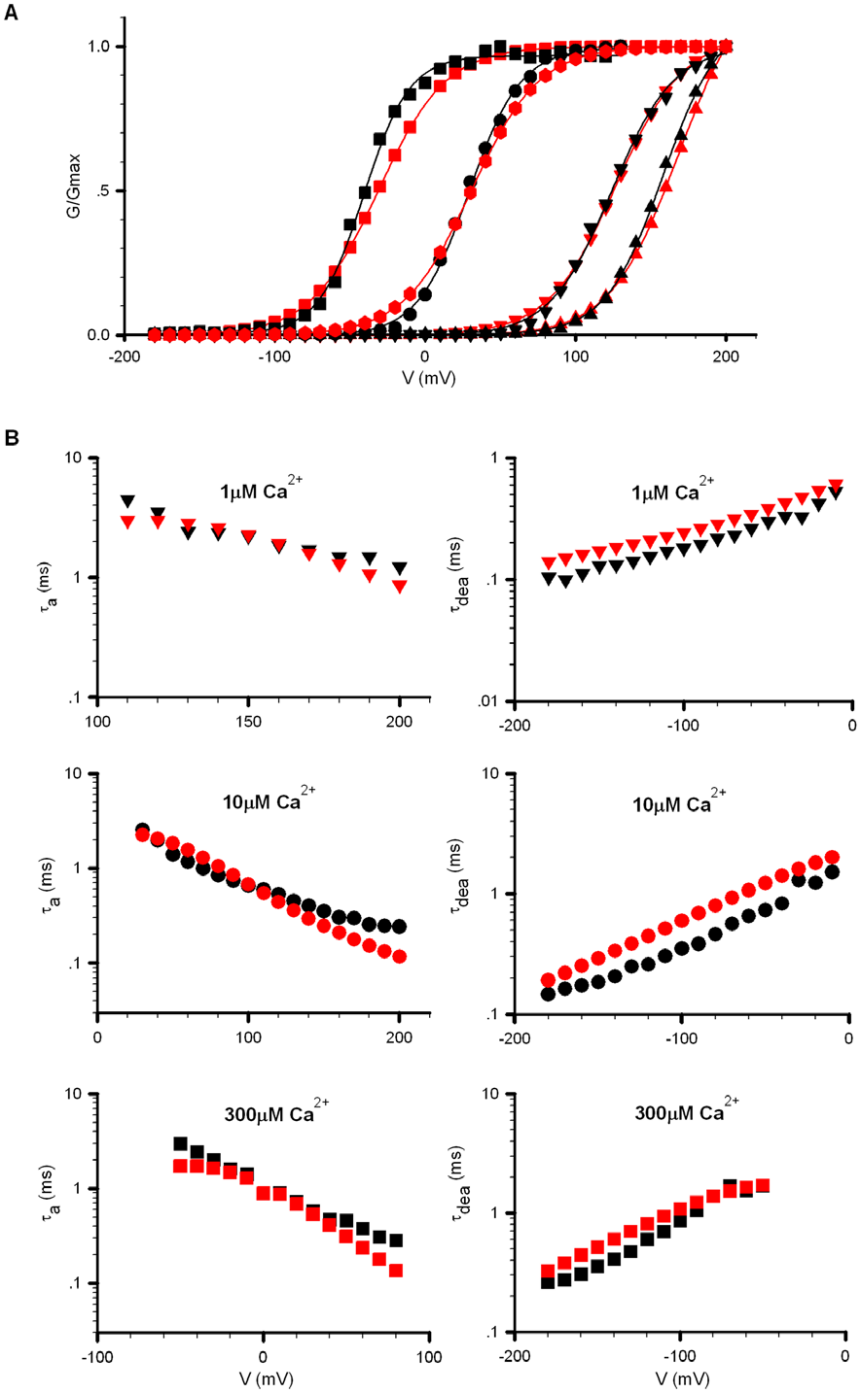
Comparison of kinetic characteristics between simulation data and target data. (A) The G-V curves of BK channels were plotted for data and best-fit, in the presence of 1, 10 and 300 µM Ca^2+^, respectively. (B) Time constants of activation (Left) and deactivation (Right) of BK channels were plotted for data and best-fit, in the presence of 1, 10 and 300 µM Ca^2+^, respectively. Here data are in black and fits in red.

**Figure 6:**
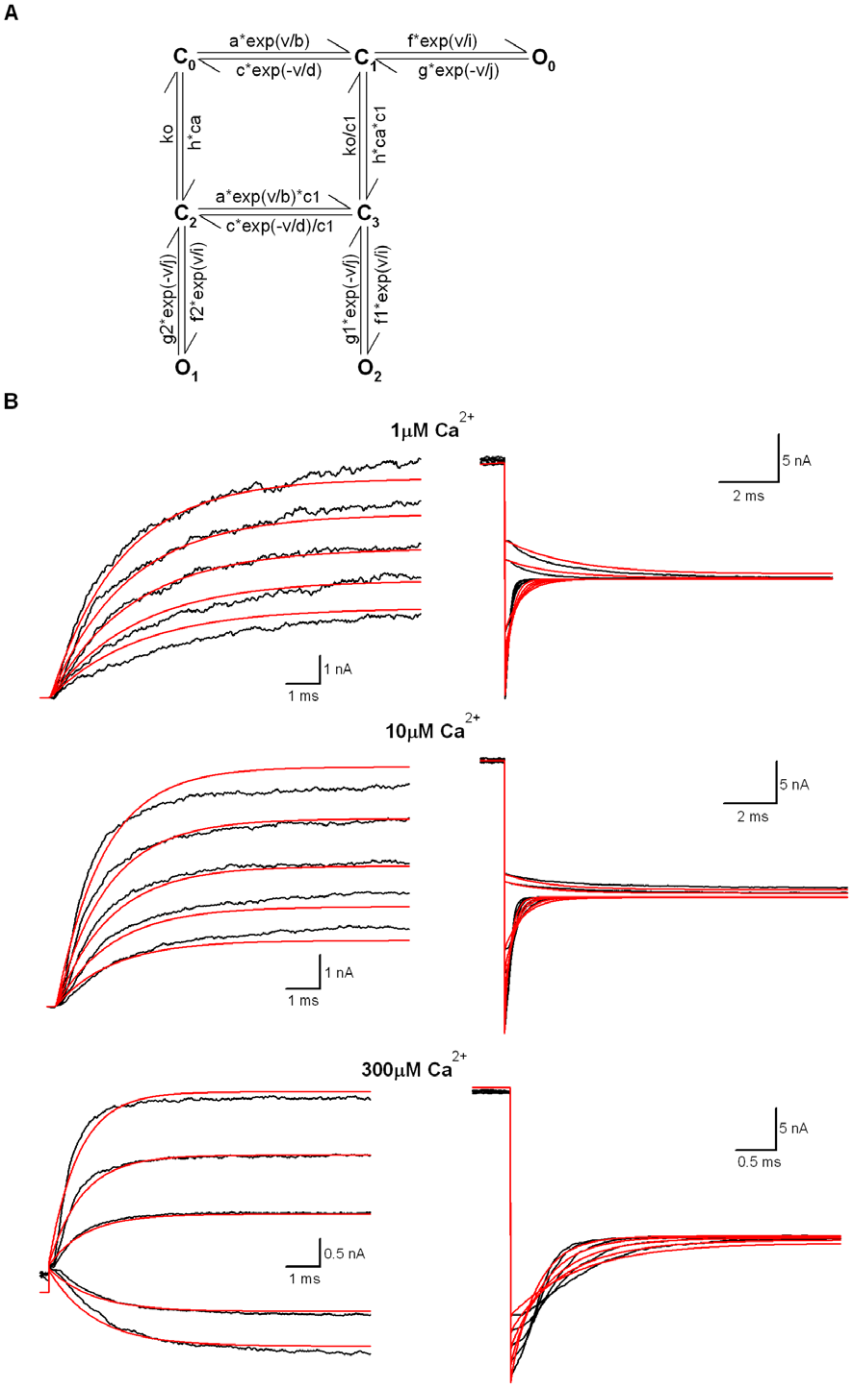
A 7-state BK model used for describing the same currents in **Figure 4** **.** (A) A 7-state BK model simplified from the 50-state MWC model of BK channels. (B) The parameters of the 7-state model were: a = 342.37 s^−1^; b = 67.90 mV; c = 56821.90 s^−1^; d = 168.47 mV; f = 10.21 s^−1^; i = 232.72 mV; g = 71130.28 s^−1^; j = 121.54 mV; h = 1330.129 s^−1^ M^−1^; c1 = 3.97; k_O_ = 562.23 M; f1 = 10^5^ s^−1^; g1 = 1323.36 s^−1^; f2 = 10.02 s^−1^; g2 = 99575.30 s^−1^. The score σ^2^ is 84.99. Compared with the 10-state MWC model, we have 
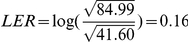
.

Until now, all the examples dealt with PSO-GSS were derived from the kinetic simulations created manually in advance, i.e., the data used so far were ideal ones. However, the practical data always contained several kinds of noises, such as thermal noise, gating noise and capacitive noise. It was important to know the antinoise ability of algorithm. Therefore, the macroscopic currents of BK channels recorded from *oocytes* were used for this purpose. The model C_5_-O_5_ shown in Figure 2A was used for fitting the experimental data. The practical data brought forth three problems: capacitive noise; channel reopen; delayed current. The capacitive noise came from voltage steps. The channel reopen might come from recovery of Ca^2+^ block at higher voltages or the miss match between model and data. The third one was due to the miss match between model and data. Thus, we substituted all the points with a straight line that would never be counted during run to reduce the effects coming from capacitive noise and reopen. In Figure 4A–B, the goodness of fit seemed to be good for currents only. The G-V curves and time constants of model channels were plotted in Figure 5A–B, respectively, indicating that the goodness of fit is good again. The global best-fit parameters by PSO-GSS shown in Table 1 was mostly consistent with that previously calculated by Horrigan et al. [7], indicating that the global fitting is a better way for building modeling. It was known that the same data can be explained by several different models. Fitness of model matching currents would have an impact on precisely describing the action potentials of cells. Thus, it was meaningful to distinguish which model provides the better fitness. In Figure 6A, a 7-state model differing from 10-state MWC model was used to fit BK currents as shown in Figure 5A. Obviously, it produced a worse fit by eye or LER = 0.16 (Figure 6B).

**Table 1:** Parameters Used for Current Simulations.

Activation parameters	Range defined in Cox et al. [8]	Value used for current simulations
L(0)	1647–2029	1355.59
Q	1.35-1.40e	1.18e
K_C_	8.68–11.0 µM	14.10 µM
K_O_	1.04–1.10 µM	1.57 µM
C_0_→O_0_	1.8–2.39 s^−1^	0.077 s^−1^
C_1_→O_1_	5.0–7.0 s^−1^	20.11 s^−1^
C_2_→O_2_	29–40 s^−1^	7.10 s^−1^
C_3_→O_3_	130–295 s^−1^	360.12 s^−1^
C_4_→O_4_	300 s^−1^	840.33 s^−1^
q_0_	0.71-0.73e	0.89e
O_0_→C_0_	3612–3936 s^−1^	130.45 s^−1^
O_1_→C_1_	1076–1338 s^−1^	3624.34 s^−1^
O_2_→C_2_	659–974 s^−1^	135.39 s^−1^
O_3_→C_3_	480–490 s^−1^	725.92 s^−1^
O_4_→C_4_	92–126 s^−1^	179.14 s^−1^
q_c_	−0.64 to −0.67e	−1.63e
Ca^2+^ on rates per site	10^9^ M^−1^ s^−1^	10^9^ M^−1^ s^−1^

## Discussion

In this study, we have developed a new approach based on the PSO-GSS algorithm for kinetic analysis of macroscopic currents of ion channels. The approach is applicable to data obtained with arbitrary voltage protocols. It also allows for global fitting of current traces with models of arbitrary topology and complexity. The fitting typically takes a few minutes for models at a reasonable size. It is also applicable for more complicated models (e.g. 13 parameters) with a larger datasets, though the fitting takes a longer time, e.g., ∼10 hours on a desktop computer with a single AMD Phenom 3.2 GHz CPU. Because calculation of initial probabilities of all the states is automatic before starting parameter space search by default, we thus suggest that starting position of each current trace should be selected at steady states. Additionally, the capacitive noises of raw data were eliminated before starting a fit.

The method in this study shows high efficiency in calculation mostly due to the PSO-GSS algorithm with the corresponding estimation in initial values. Is this estimation really necessary? Answer is certainly positive. Our results indicated that the number of generation cost for the simpler model was shortened at least to half, using initial estimations. Additionally, it is not only easy to get a good approximation as initial guess, but also investigators usually want to use his own initial guess with a different boundary factor at beginning, and to make some changes to parameters during calculation, that let it become more friendly. When using the default values, it is better to make a change on the exponential factor from 0.5 to 10, which may create a very large number difficult to calculate at the higher voltages. Additionally, we found that either channel count N_C_ or single-channel conductance could be crucial for global fitting, as a bad approximation of N_C_ may severely affect actual probability of each state. Fortunately, good approximation of N_C_ can easily obtain after measuring the saturated currents of channels.

For the complicated models, e.g., BK-like or Na_V_-like models with huge amounts of data, our approach needs a relatively longer time to converge to the target values with an average error of 1%. Considering the stochastic property of PSO, we tested the repeatability of convergence of the algorithm. Our data showed that it had good repeatability of convergence for the BK-like models with 11-parameter (n = 3), but disaccord for the Na_V_-like models with 13 parameters. In Figure S3 A, one score declined to 10^−6^, two to 10^−5^ and three to 10^−4^. With six tests, the mean errors derived from 12 parameters except N_C_ are ∼20% (n = 3), ∼10% (n = 2) and ∼1% (n = 1), respectively; the minimal mean error is less than 1% and the maximal mean error is more than 60% (Figure S3 B). However, fixing the constraint factor c = 4.436203 (target value), we completely eliminated the instability of convergence (n = 3), suggesting that it is coming from the constraint i = ruj/(swgc^8^) with a big factor c^8^.

Macroscopic responses of ion channels are less rich in kinetics than single-channel events. Thus, they allow fewer parameters of the model to be identified. To improve the issue, we added constraints on the model. Our tests suggest that the incorporation of such constraints greatly improves the identifibility of complex models. For all the examples considered here, our algorithm successfully recovers the parameters of the models from data of activation, deactivation, inactivation, steady-state inactivation and recovery of inactivation.

## Materials and Methods

### Simulation environment

PSO-GSS algorithm written by the Visual C++ language is running on a desktop computer with a AMD Phenom 3.2 GHz CPU, Windows-XP system. Algorithm will automatically calculate the starting probabilities of each state before fitting. The established model can be used to further calculate action potentials in a model cell [6]. All data in a format of ABF were analyzed using the software Clampfit (MDC/Axon Instruments, USA).

### Particle swarm optimization-golden section search algorithm

Population-based random search method is a kind of stochastic optimization method that can be applied to a wide variety of problems (whether it is linear or nonlinear, continuous or discrete, differentiable or not). It is especially suitable for the traditional optimization problems difficult to be solved, such as those with no analytical solution, multi-modal and multi-objective criterions and large variable dimensions. The method has been successfully applied to a broad range of science and engineering problems, such as, task matching and scheduling [10], capacitated multipoint network design [11], nonlinear controller design of power system [12], distributed database management [13], large-scale circuit design [14], [15], military tactical planning [16], etc.

PSO algorithm [17], [18] is a typical population-based heuristic global stochastic optimization technique introduced by Kennedy and Eberhart in 1995. Its basic idea is based on the simulation of simplified animal social behaviors such as fish schooling, bird flocking, etc. In classic PSO, a population of random particles (solutions) is initialized. The optimal particle (solution) is then found through continuous updating in generations. In every generation, particles are updated by tracking two ‘extremums’. One is the optimal value found by the current particle so far, and the other is the optimal value found by all the particles so far. The former one denotes the local optimal information, and the latter one represents the global optimal information. Combining them with the inertia information of the particle, which contains its historical information, the particles move towards the optimal value gradually in the search space.

**Figure 7:**
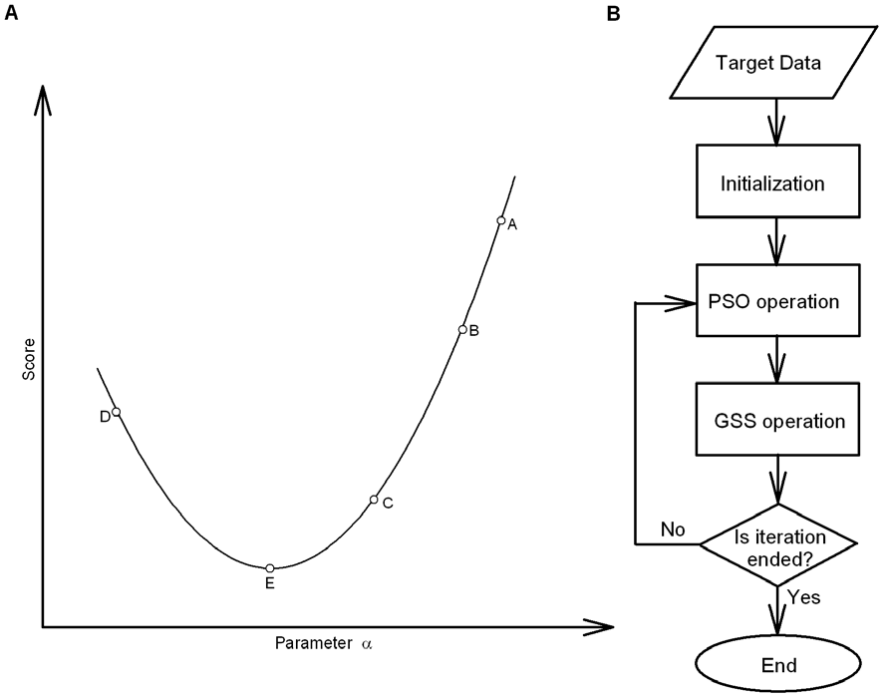
Schematic diagram for GSS and PSO. (A) A schematic drawing for golden section search algorithm (GSS). (B) A flow graph for the PSO-GSS algorithm.

In the present work, we used an adaptive inertial weighted PSO algorithm with a genetic operator [19], [20]. The population was sorted according to each individual value of the cost function in Equation 2, and new generation was created using selection and crossover operators of PSO algorithm. Our simulations suggest that the kinetic fitting of ion channels follow a unimodal-type function in a local area. As a result, we applied the classic linear search procedure, golden section search (GSS) [21] for searching the optimal solutions locally. GSS is a technique using for finding the extremum of a unimodal function by successively narrowing the range of parameters. For example, assuming that the point A is the previous best parameter set and B the present best parameter set, GSS searches for the new best solution along the vector 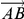 (Figure 7A). In the succeeding search, if the point C is better than B while the point D worse than C, the local optimal solution E will be identified between B and D. The incorporation of the GSS technique improves the efficiency and precision of the PSO algorithm. Figure 7B shows the flow diagram of the whole search procedure. First, the PSO is initialized to set parameters and operators. Then PSO is started for one generation and the resulting optimal value is sent to the GSS for further local optimization. Next, the solution is tested for the stop criterion, which is normally defined as a constant generation maximum. If the stop criterion is not satisfied, the algorithm loops back to PSO and continues for the next generation; otherwise, the optimization stops and the solution is taken as the optimal value. During iterations, the best solution was independently saved for each generation. Once a better one was found, it was used to replace the previous one, which was considered as a displacement in the parameter space.

Our particle swarm optimization in combination with a golden section search algorithm (GSS) is much faster than that described by Gurkiewicz and Korngreen [3], since we have taken three measures to improve the searching efficiency of PSO-GSS algorthm:

1. PSO and GSS work interactively in each generation so that the global and local information can be integrated.
2. Real number coding instead of binary coding in GA is employed in PSO to improve the searching accuracy.
3. Both PSO and GSS require no gradient information.

The pseudocode of the PSO-GSS algorithm can be found in the Supporting file.S1

PSO-GSS searches the true parameter values of models in parameter spaces. Given the larger spaces, it often costs the longer computational time. Given a starting value of parameters and a boundary factor, we can define a window ranging from starting value/boundary factor to starting value* boundary factor. Without prior knowledge, we can define the default window ranging from 0.005 and 50, setting the initial values  = 0.5 and the boundary factors = 100. In this study, the unit is ms^−1^ for the preexponential factor of rate constants *k*, and mV^−1^ for the exponential factor of rate constants *k*. In other words, a default range is between 0.005 and 50 ms^−1^ for preexponential factor, and between 0.005 and 50 mV^−1^ for exponential factor. Virtually, those ranges are adequate for most models in practice. The channel population Nc can be selected manually, e.g. Nc = 250 pS. The state occupancy of a model is calculated by Q-matrix [22].

We define the cost function σ^2^ (or score) as the relative least square error between fits and data:
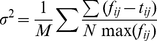
(2)Where *f* is the fit, *t* is the data, *N* is the point number of data, and *M* is the number of sweeps. Here the normalized least square error promotes the significance of data with smaller current and less point.

To rank fitness of different models, we used the Log Error Ratio(LER): LER = log(σ_A_/σ_B_), where σ_A_ and σ_B_ are the root mean squares for fitting the same data to the models A and B, respectively [3].

### Estimating the initial values of the model parameters from macroscopic currents

Stochastic search usually costs tremendous computational time in the wider ranges, especially dealing with a larger set of parameters. Additionally, some parameters may go beyond those ranges. A proper estimation of initial values can help guiding the whole searching process around the target values in order to reduce computational time as we select initial values for fitting a function to data.

We can decompose the whole models into several basic kinetic elements, because the basic kinetic course of ion-channel gating is principally composed of several element reactions: activation (C→O), deactivation (C←O), inactivation (O→I) and recovery from inactivation (O←I or C←O←I), where C, O and I are the closed, open and inactivation states, respectively. For example, the model C←→O←→I can be divided into two basic kinetic courses: C←→O and O←→I, in which the estimated values will not be far off the true ones. It is known that macroscopic currents of channels can be written as I = N*g*P_o_*(v−v_r_), where N, g, P_o_, v and v_r_ are the channel number, the single channel conductance, the open probability, the membrane potential and the reversal potential of channels, respectively. Here, the open probability P_o_ of the simplest voltage-dependent dynamic course C←→O has an analytic expression, i.e., P_o_(t) = *k_f_*/(*k_f_*+*k_b_*)*(1−exp(−(*k_f_*+*k_b_*)*t)) for activation process, and P_o_(t) = P_o_(t_0_)*exp(−(*k_f_*+*k_b_*)*t)) for deactivation process, where the forward rate constant *k_f_*(v) = a*exp(v/b) and the backward rate constant *k_b_*(v) = c*exp(−v/d). The letters a, b, c and d represent the pending parameters (Figure 1A). In other words, we always have the current I(t)∝exp(−(*k_f_*+*k_b_*)*t) in both courses. Considering two different cases or directions, i.e., the extremely positive or negative voltages, we have I(t)∝exp(−k_f_*t) when *k_f_*>*k_b_* at positive voltages or I(t)∝exp(−k_b_*t) when *k_b_*>*k_f_* at negative voltages. Fitting single exponential function to the activation currents or deactivation currents separately, we can obtain a set of values of *k_f_*(v) and *k_b_*(v), respectively. After that, we fit the expression a*exp(v/b) to *k_f_*(v) to get the parameters a and b. The same procedure can be used to get the parameters c and d. Based on these initial values, we further narrow the searching range by reducing the boundary factors to 5 or less in order to save the computational cost greatly. The whole procedure can be summarized as below:

1. Discompose the original model into a series of element reactions, such as: C→O, C←O, O→I, etc.
2. For each of the above element reactions, fit I(t)∝exp(−*k*t) in the *k* course to get a set of values of *k*(v).
3. Fit an exponential function to *k*(v) to get the pre-exponential and exponential factors.

Experientially, it is better to obtain a good approximation for the channel number N_C_ so as to roughly keep a suitable initial occupancy of states. With a good estimation on N_C_, we simply set the boundary factor = 1.5 or less. For exponential factors, we usually set them larger than 10 to avoid larger numbers appearing at extreme voltages, which may stop fitting.

### Electrophysiology

mSlo1 cRNA was prepared as we described previously [23]. 10–20 ng/µl of RNA was injected into stage IV *Xenopus* oocytes harvested 1 d before. The injected oocytes were maintained in ND96 solutions (96 mM NaCl, 2.0 mM KCl, 1.8 mM CaCl_2_, 1.0 mM MgCl_2_, and 5.0 mM HEPES, pH 7.5) supplemented with 2.5 mM sodium pyruvate, 100 U/ml penicillin, 100 mg/ml streptomycin, and 50 mg/ml gentamicin at 17°C.

Currents were recorded in inside-out patches with symmetric K^+^ solutions, and typically digitized at 10–50 kHz. In some experiments, a sampling rate of 100 or 200 kHz was used. Data were filtered at 5–20 kHz using the built-in Bessel low-pass filter in the amplifier. The extracellular solution consisted of (mM):140 potassium methanesulfonate (MES), 20 KOH, 10 HEPES, and 2 MgCl2, pH 7.0. The intracellular solution contained 140 potassium MES, 20 KOH, 10 HEPES, pH 7.0, with Ca2+ buffered by either 5 EGTA (for nominally 0–1 µM Ca^2+^) or 5 HEDTA (for 4–10 µM Ca^2+^). For 60 µM–5 mM Ca^2+^, no buffer was used. Free [Ca^2+^] was calculated using the EGTAETC program (E. McCleskey, Vollum Institute, Portland, OR). Calibration of [Ca^2+^] was as we previously described [23]. Experiments were done at room temperature (21–24°C). All the chemicals were purchased from Sigma-Aldrich.

Data were analyzed with ClampFit (MDC/Axon Instruments, USA). G-V curves of activation were fit with Boltzmann equation: G/G_max_ = (1+exp((V−V_50_)/κ)^−1^, where V_50_ is the voltage at which the conductance (G) is half the maximum conductance (G_max_), and κ determines the slope of the curve.

## Supporting Information

Figure S1 **Fit a nine-parameter voltage-dependent C-O-I model to the target current traces.** (A) A nive-parameter Markov model consisting of a closed state, an open state and an inactivation state labeled with the letter C, O and I, respectively. The parameters to be fitted are similar to that we described in Fig. 1(A). (B–C) See the description in Fig. 1B–C. (D–E) In this model, the target parameters a = 0.001 ms-1, b = 50 mV, c = 0.081 ms-1, d = 90 mV, e = 0.015 ms-1, f = 200 mV, g = 0.007 ms-1 and h = 30 mV; the reversal potential of channels Vr = 0 mV; the single-channel conductance G = 250 pS and the channel count N_C_ = 1. The empty circles denote target currents and the solid lines represent fitted currents. The voltage protocols are placed below each of current traces.
(TIF)

Figure S2 **Fit a five-parameter Kv-like C4-O model to the target current traces.** (A) A five-parameter Markov model consisting of four closed states and an open state labeled with the letter C and O, respectively. The parameters to be fitted is similar to that we described in Fig. 1(A). (B–C) See the description in Fig. 1B–C. (D) In this model, the target parameters a = 0.0414 ms-1, b = 22 mV, c = 0.0072 ms-1 and d = 45 mV; the reversal potential of channels Vr = 0 mV; the single-channel conductance G = 250 pS and the channel count N_C_ = 1. The empty circles denote target currents and the solid lines represent fitted currents. The voltage protocols are placed below each of current traces.
(TIF)

Figure S3 **Repeatability of convergence of the fit for NaV-like channels.** (A) Converging behaviors of PSO-GSS algorithm for 13-parameter NaV-like channel model. (B) Summary on the mean errors of 12 parameters except N_C_.
(TIF)

Table S1 **A Comparison between PSO-GSS and GA+PrAxis.**
(DOC)

Supporting File S1 (DOC)
