# Supplementary figures and images for: Optimal Estimation of Ion-Channel Kinetics from Macroscopic Currents
Source: PLoS One. 2012 Apr 20;7(4):e35208. doi: 10.1371/journal.pone.0035208 (PMC3335051; doi:10.1371/journal.pone.0035208)

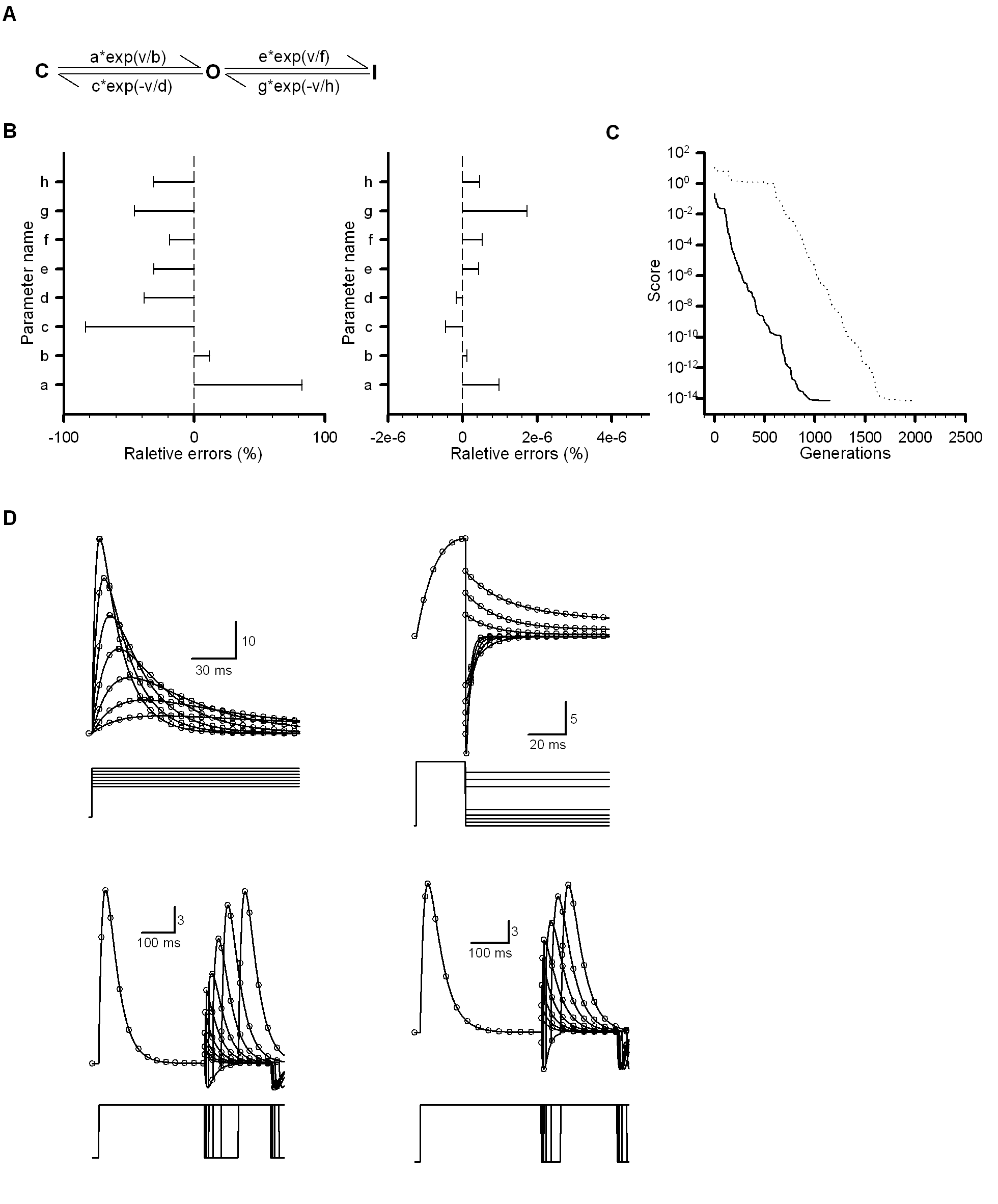

Supplement: Figure S1 — Fit a nine-parameter voltage-dependent C-O-I model to the target current traces. (A) A nive-parameter Markov model consisting of a closed state, an open state and an inactivation state labeled with the letter C, O and I, respectively. The parameters to be fitted are similar to that we described in Fig. 1(A). (B–C) See the description in Fig. 1B–C. (D–E) In this model, the target parameters a = 0.001 ms-1, b = 50 mV, c = 0.081 ms-1, d = 90 mV, e = 0.015 ms-1, f = 200 mV, g = 0.007 ms-1 and h = 30 mV; the reversal potential of channels Vr = 0 mV; the single-channel conductance G = 250 pS and the channel count NC = 1. The empty circles denote target currents and the solid lines represent fitted currents. The voltage protocols are placed below each of current traces. (TIF) [file pone.0035208.s001.tif]

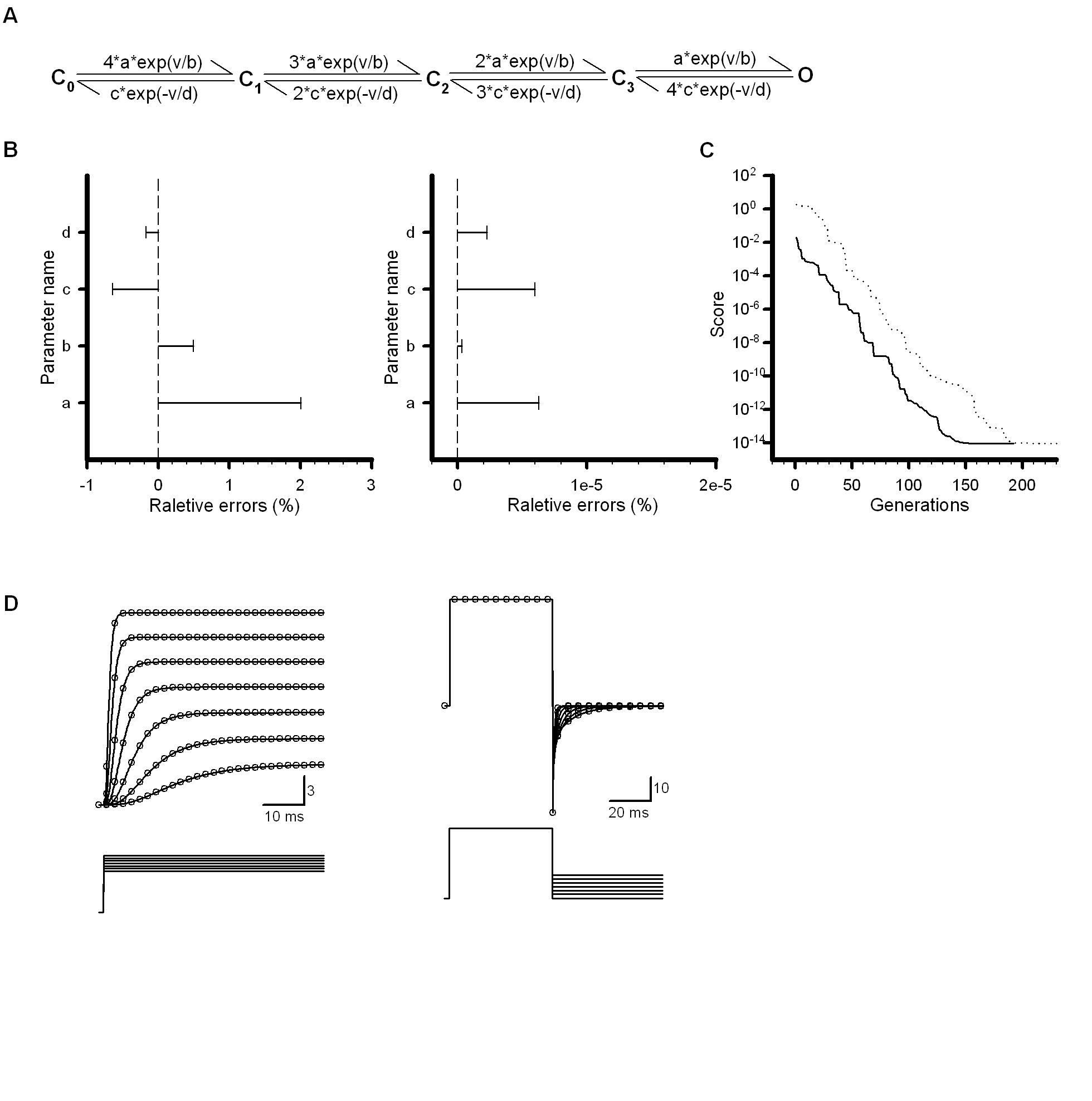

Supplement: Figure S2 — Fit a five-parameter Kv-like C4-O model to the target current traces. (A) A five-parameter Markov model consisting of four closed states and an open state labeled with the letter C and O, respectively. The parameters to be fitted is similar to that we described in Fig. 1(A). (B–C) See the description in Fig. 1B–C. (D) In this model, the target parameters a = 0.0414 ms-1, b = 22 mV, c = 0.0072 ms-1 and d = 45 mV; the reversal potential of channels Vr = 0 mV; the single-channel conductance G = 250 pS and the channel count NC = 1. The empty circles denote target currents and the solid lines represent fitted currents. The voltage protocols are placed below each of current traces. (TIF) [file pone.0035208.s002.tif]

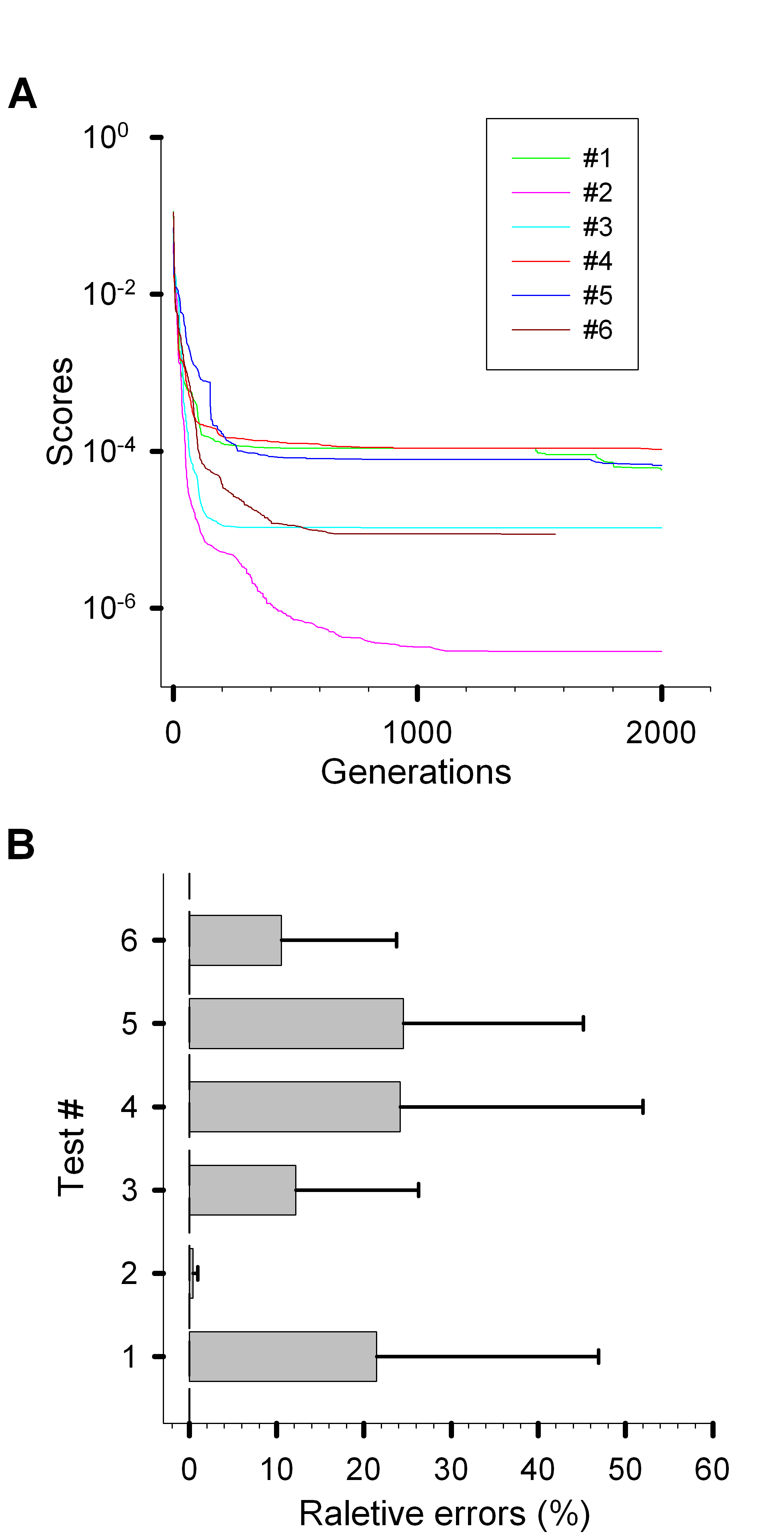

Supplement: Figure S3 — Repeatability of convergence of the fit for NaV-like channels. (A) Converging behaviors of PSO-GSS algorithm for 13-parameter NaV-like channel model. (B) Summary on the mean errors of 12 parameters except NC. (TIF) [file pone.0035208.s003.tif]
