# Supplementary material for: Optimal Estimation of Ion-Channel Kinetics from Macroscopic Currents
Source: PLoS One. 2012 Apr 20;7(4):e35208. doi: 10.1371/journal.pone.0035208 (PMC3335051; doi:10.1371/journal.pone.0035208)
Supplement: Table S1 — A Comparison between PSO-GSS and GA+PrAxis. (DOC) [file pone.0035208.s004.doc]

**Table S1. A Comparison between PSO-GSS and GA+PrAxis**

|  | Kinetic Model | Computer | Time | Error |
| --- | --- | --- | --- | --- |
| PSO-GSS | 10-state/20-parameter | AMD Phenom 3.2 GHz clock speed (One core) | ~6 hours | 1% |
|  | 4-state/6-parameter | AMD Phenom 3.2 GHz clock speed (One core) | ~2 minutes |  |
| GA+PrAxis | 20-parameter | 10 Pentium 4 computers with a 3 GHz clock speed | more than a week | 5% |
|  | a simple model with a small amount of data | Pentium 4 with a 3 GHz clock speed | 2 hours |  |
|  |  | 10 Pentium 4 computers with a 3 GHz clock speed | less than 20 minutes |  |
