# Supplementary material for: Optimal Estimation of Ion-Channel Kinetics from Macroscopic Currents
Source: PLoS One. 2012 Apr 20;7(4):e35208. doi: 10.1371/journal.pone.0035208 (PMC3335051; doi:10.1371/journal.pone.0035208)
Supplement: Supporting File S1 — (DOC) [file pone.0035208.s005.doc]

**The pseudocode of the PSO-GSS algorithm**

PSO :

1 for i = 1 to paramnum do

2 left _i_ = InitVal _i_ ÷ Boundary _i_

3 right _i_ = InitVal _i_ × Boundary _i_

4 if ( left _i_ > right _i_ ) then

5 Swap( left _i_ , right _i_ )

6 fi

7 od

8 for i = 1 to population do

9 for j = 1 to paramnum do

10 PositionParam _i,j_ = ( right _j_ - left _j_ ) × Random (0,1)

11 PositionBetterparam _i,j_ = PositionParam _i,j_

12 PositionSpeed _i,j_ = speedmax _j_ × Random (0,1)

13 od

14 PositionScore _i_ = CalcFitness( PositionParam _i_ )

15 PositionBetterscore _i_ = PositionScore _i_

16 od

17 BestpositionScore = PositionScore _1_

18 for i = 1 to paramnum do

19 BestpositionParam _i_ = PositionParam _1_,_i_

20 od

21 for i = 2 to population do

22 if ( BestpositionScore > PositionScore _i_ ) then

23 for j = 1 to paramnum do

24 BestpositionParam _j_ = PositionParam _i,j_

25 od

26 fi

27 od

28 LastbestpositionScore = BestpositionScore

29 for i = 1 to paramnum do

30 LastbestpositionParam _i_ = BestpositionParam _i_

31 od

32 for i = 1 to iteration do

33 for j = 1 to population÷2 do

34 k = 2 + ( paramnum-3 ) × Random(0,1)

35 for m = 1 to k do

36 Swap( PositionParam _j,m_ , PositionParam _population÷2+j,m_ )

37 od

38 od

39 for j = 1 to population do

40 for k = 1 to paramnum do

41 PositionSpeed _j,k_ = w × PositionSpeed _j,k_ + weight1 × Random (0,1) × ( PositionBetterparam _j,k_ - PositionParam _j,k_ ) + weight2×Random (0,1)×( BestpositionParam _k_ – PositionParam _j,k_ )

42 PositionParam _j,k_ = PositionParam _j,k_ + PositionSpeed _j,k_

43 od

44 PositionScore _j_ = CalcFitness( PositionParam _j_ )

45 if ( BestpositionScore > PositionScore _j_ ) then

46 BestpositionScore = PositionScore _j_

47 for k = 1 to paramnum do

48 BestpositionParam _k_ = PositionParam _j,k_

49 od

50 fi

51 if ( PositionBetterscore _j_ > PositionScore _j_ ) then

52 PositionBetterscore _j_ = PositionScore _j_

53 for k = 1 to paramnum do

54 PositionBetterparam _j,k_ = PositionParam _j,k_

55 od

56 fi

57 od

58 GSS ( BestposotionParam , BestpositionScore,LastbestpositionParam, LastbestpositionScore )

59 LastbestpositionScore = BestpositionScore

60 for j = 1 to paramnum do

61 LastbestpositionParam _j_ = BestposotionParam _j_

62 od

63 od

GSS:

GSS ( BestposotionParam , BestpositionScore,LastbestpositionParam, LastbestpositionScore )

1 leftpositionScore = LastbestpositionScore

2 rightpositionScore = BestpositionScore

3 for i = 1 to paramnum do

4 leftposotionParam _i_ = LastbestpositionParam _i_

5 rightposotionParam _i_ = BestposotionParam _i_

6 step _i_ = rightposotionParam _i_ - leftposotionParam _i_

7 od

8 do

9 for i = 1 to paramnum do

10 NextposotionParam _i_ = rightposotionParam _i_ + step _i_

11 od

12 NextposotionScore = CalcFitness(NextposotionParam)

13 if (NextposotionScore < rightpositionScore ) then

14 for i = 1 to paramnum do

15 leftposotionParam _i_ = rightposotionParam _i_

16 rightposotionParam _i_ = NextposotionParam _i_

17 od

18 leftpositionScore = rightpositionScore

19 rightpositionScore = NextposotionScore

20 fi

21 until (NextposotionScore > rightpositionScore )

22 for i = 1 to paramnum do

23 leftGoldenposotion _i_ = leftposotionParam _i_ + 0.382 * (NextposotionParam _i_ - leftposotionParam _i_)

24 rightGoldenposotion _i_ = leftposotionParam _i_ + 0.618 * (NextposotionParam _i_ - leftposotionParam _i_)

25 od

26 leftGoldenScore = CalcFitness(leftGoldenposotion)

27 rightGoldenScore = CalcFitness(rightGoldenposotion)

28 for i = 1 to iterat do

29 if (leftGoldenScore < rightGoldenScore ) then

30 for j = 1 to paramnum do

31 NextposotionParam _j_ = rightGoldenposotion _j_

32 rightGoldenposotion _j_ = leftGoldenposotion _j_

33 leftGoldenposotion _j_ = leftposotionParam _j_ + 0.382 * (NextposotionParam _j_ - leftposotionParam _j_)

34 od

35 NextposotionScore = rightGoldenScore

36 rightGoldenScore = leftGoldenScore

37 leftGoldenScore = CalcFitness(leftGoldenposotion)

38 else

39 for j = 1 to paramnum do

40 leftposotionParam _i_ = leftGoldenposotion _j_

41 leftGoldenposotion _j_ = rightGoldenposotion _j_

42 rightGoldenposotion _j_ = leftposotionParam _j_ + 0.618 * (NextposotionParam _j_ - leftposotionParam _j_)

43 od

44 leftpositionScore = leftGoldenScore

45 leftGoldenScore = rightGoldenScore

46 rightGoldenScore = CalcFitness(rightGoldenposotion)

47 fi

48 od

49 for i = 1 to paramnum do

50 leftGoldenposotion _i_ = (NextposotionParam _j_ + leftposotionParam _i_ ) ÷ 2

51 od

52 leftGoldenScore = CalcFitness(leftGoldenposotion)

53 if (leftGoldenScore < BestpositionScore ) then

54 BestpositionScore = leftGoldenScore

55 for i = 1 to paramnum do

56 BestposotionParam _i_ = leftGoldenposotion _i_

57 od

58 fi
